# A comparison of alternative 60-mer probe designs in an in-situ synthesized oligonucleotide microarray

**DOI:** 10.1186/1471-2164-7-72

**Published:** 2006-04-04

**Authors:** Danielle L Leiske, Anis Karimpour-Fard, Patrick S Hume, Benjamin D Fairbanks, Ryan T Gill

**Affiliations:** 1 Chemical and Biological Engineering, University of Colorado, Boulder, Colorado, USA; 2 Computational Pharmacology, University of Colorado School of Medicine, Denver, Colorado, USA

## Abstract

**Background:**

DNA microarrays have proven powerful for functional genomics studies. Several technologies exist for the generation of whole-genome arrays. It is well documented that 25mer probes directed against different regions of the same gene produce variable signal intensity values. However, the extent to which this is true for probes of greater length (60mers) is not well characterized. Moreover, this information has not previously been reported for whole-genome arrays designed against bacteria, whose genomes may differ substantially in characteristics directly affecting microarray performance.

**Results:**

We report here an analysis of alternative 60mer probe designs for an in-situ synthesized oligonucleotide array for the GC rich, β-proteobacterium *Burkholderia cenocepacia*. Probes were designed using the ArrayOligoSel3.5 software package and whole-genome microarrays synthesized by Agilent, Inc. using their in-situ, ink-jet technology platform. We first validated the quality of the microarrays as demonstrated by an average signal to noise ratio of >1000. Next, we determined that the variance of replicate probes (1178 total probes examined) of identical sequence was 3.8% whereas the variance of alternative probes (558 total alternative probes examined) designs was 9.5%. We determined that depending upon the definition, about 2.4% of replicate and 7.8% of alternative probes produced outlier conclusions. Finally, we determined none of the probe design subscores (GC content, internal repeat, binding energy and self annealment) produced by ArrayOligoSel3.5 were predictive or probes that produced outlier signals.

**Conclusion:**

Our analysis demonstrated that the use of multiple probes per target sequence is not essential for in-situ synthesized 60mer oligonucleotide arrays designed against bacteria. Although probes producing outlier signals were identified, the use of ratios results in less than 10% of such outlier conclusions. We also determined that several different measures commonly utilized in probe design were not predictive of outlier probes.

## Background

DNA microarray technology has proven valuable for improving the efficiency of traditional approaches for studying genome structure and function [1,2]. Microarray technology allows for the simultaneous examination of thousands of genes for applications ranging from transcriptional profiling (used to gain insight into gene function) [2] to genomic comparisons (including evolutionary classification of bacterial strains) [3-5]. Although microarrays have proven useful, further developments are required to standardize physical and experimental designs [6]. In particular, important issues remaining include how to account for and normalize data for probe-to-probe variations, how to address differences between laboratories (sample preparation and data analysis), and how to determine whether the array is reporting biologically accurate results [7-10]. Probe to probe variation is especially important when using arrays to look for copy number and/or presence or absence of genes in comparative studies. That is, probes of variable sequence all designed against the same gene can have different affinities for the target sequence, bringing into question the number of probes required for each target and how this issue changes as a function of probe length and characteristics of the target organism's genome. Here, we describe an analysis of the probe to probe variations for an in situ synthesized oligonucleotide array comprised of 60mers designed against the recently sequenced genome of *Burkholderia cenocepacia *J2315. *B. cenocepacia *is a particularly useful model organism for the studies described here. Specifically, its genome is GC rich (66.9%), which challenges the design (uniqueness) and application (hybridization stringency) of whole-genome microarrays in this organism.

As array technology grows in popularity, issues regarding probe design and repeatability are beginning to be addressed by things such as improved construction methods and standardization of techniques. New array designs such as Agilent's ink-jet spotted arrays, [11] have made custom arrays simple to design and construct. This enhanced construction combined with ongoing genome sequencing projects (as of March 1, 2006, 1,951 genome sequences are either complete or in the process [12]) has made microarray technology applicable to any well-studied organism. However, the increased demand for custom arrays illuminates the challenge that lies within the area of probe design. It is well documented that probe specificity and sensitivity depend on multiple factors including uniqueness, GC content, steric hindrance, and distance from the 3' end of the ORF [13,14]. Additionally, it has been observed that probes with different sequences designed for the same gene yield different affinities for targets [14-16]. Thermodynamic models of probes have been created in an attempt to predict the performance [17]. However, theoretical prediction has thus far proven to be a difficult task and often requires additional experimental data [14].

Analysis concentrating on the effect of probe sequence on gene expression data has been explored, but the majority of published data has focused on 25mer probes utilized on Affymetrix chips. Due to the Affymetrix platform design as single channel arrays with short probes, data analysis requires sophisticated algorithms comparing perfect match and mismatch probes in an attempt to account for non-specific binding and probe affinity [18]. This study will explore alternative probe designs for a custom 60mer Agilent ink-jet spotted array that is amenable to competitive hybridization studies (dual channel). As such, data analysis methods are based on ratios of competing fluorescence, which is expected to minimize the effects of individual probe affinity [11]. In addition, longer probes have been shown to decrease non-specific binding and increase overall probe affinity [11,14].

We designed an oligonucleotide array comprised of 8400 probes complimentary to the recently sequenced genome of *B. cenocepacia *J2315, an opportunistic pathogen of particular importance to cystic fibrosis research [19,20]. On the array, 9–15 replicates of a primary probe (identified by the probe design program, ArrayOligoSel3.5) and 4–5 alternative probes with unique sequences were included for a set of 117 genes (a total of 1736 probes). We will report on the quality of the array as a whole as defined by the overall microarray signal to noise ratio and reproducibility between array replicates. We will describe signal variation of primary probe replicates and alternative probes and discuss the identification of probes that produced outlier signals or signal ratios as defined by a variety of different criteria. Finally, we will report on the ability of different probe design subscores to predict poor probe performance.

## Results

The overall objective of this study was to examine the importance of having multiple probes directed against the same target for an in situ synthesized 60mer oligonucleotide array. We will first discuss the overall quality of the oligonucleotide array, followed with an analysis of primary and alternative probe reproducibility, and conclude with a presentation of outliers. As seen in Table 1, this array includes 1178 replicate probes to examine inter-probe ("natural") variance and 558 alternative probe designs to explore intra-probe variance.

### Overall quality of custom oligonucleotide array

**Figure 1:**
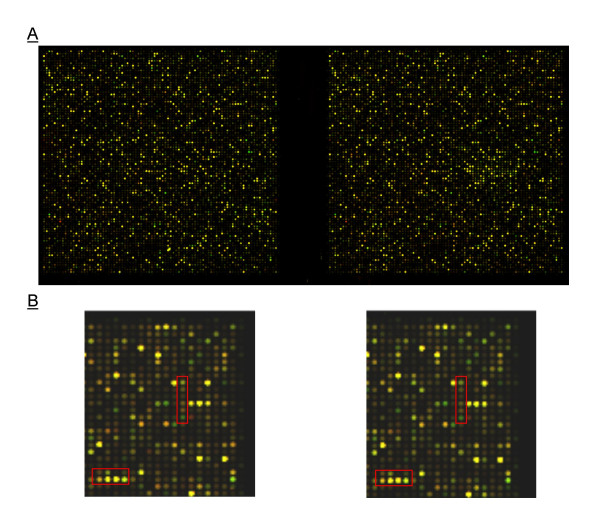
*Images of Replicate Arrays: *(A) Replicate *B. cepacia *arrays have clean, uniform spots with little background noise. (B) A closer examination of one section reveals that, qualitatively, Cy3/Cy5 ratios and relative signal intensity are the same for both arrays.

Prior to examining inter- and intra-probe variance, it was important to first examine the overall array quality as a possible source of variance within our studies. As shown in figure 1, both replicates of the array show similar fluorescence ratios for the same probes, a high signal to noise ratio, and consistent spot morphology. Replicates were significantly correlated (p = 8.07 × 10^-7^). An additional measure of overall array quality is the average signal to noise ratio (S/N = (intensity_spot _- intensity_background_)/σ_background_). For a normal distribution, a value for S/N greater than 2 indicates that the signal is significantly different than the background [21]. More generally, S/N ratios greater than 10 are considered indicative of high quality arrays [8]. The average S/N for these arrays was 1672 with a range of 4–32,000 for all probes. Thus, the overall reproducibility and quality of this custom designed oligonucleotide array is a minimal consideration in our further analysis. Rather, we are able to focus specifically on the determination of probe variation within an array for probes of identical sequence or alternative sequence directed against the same target. This issue is of particular importance not only in studies of gene expression but also gene copy number (and/or presence/absence) where differences in probe affinities are a primary concern [14-16].

### Primary probe reproducibility

We designed this array to contain up to 15 replicates of the same probe (primary probe) for 117 genes (these genes were chosen as described in materials and methods). This allowed us to i) determine if the location of the probe within the microarray had any effect on reported signal intensity values and ratios and ii) obtain relevant statistics for comparisons with alternative probes. Overall, the primary probes exhibited excellent reproducibility. Chauvenet's Criterion, used to identify outliers, distinguishes an acceptable range for data points based on the mean and standard deviation of the group. The number of measurements in the sample set defines how many standard deviations from the mean are acceptable; the larger the set the larger (and less stringent) the acceptable range. For this study the number of acceptable standard deviations ranged from 1.65–2.125 for group sizes ranging from 5–15 [22]. Using Chauvenet's Criterion, 45 of 117 sets of primary probes included one probe that was defined as an outlier in signal intensity for either heat shock, pre-heat shock, or both conditions. However, of the total number of primary probes, only 3.82% (45 out of 1178) were defined as outliers, which is well within a 95% confidence interval.

**Figure 2:**
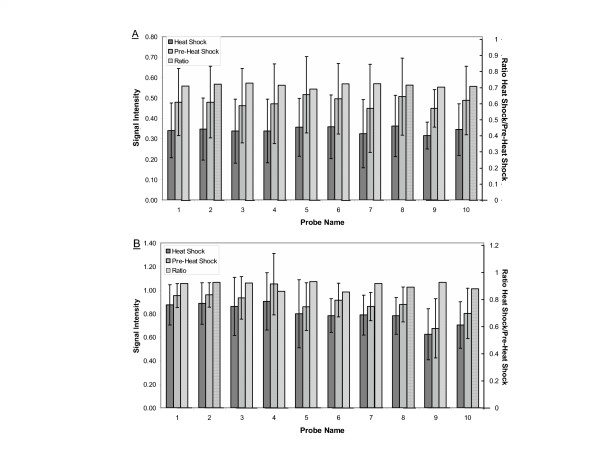
*Primary Probe Sets*. Signal intensity and heat shock/pre-heat shock ratios for primary probe replicates. Heat shock/pre-heat shock ratios between 0.7 and 1.5 exhibit no significant change in gene expression. Genes with ratios above 1.5 are up-regulated and genes below 0.7 are down-regulated. (A) The primary probe replicates for BCAL0007 identified no outliers, signal intensity and ratios are consistent for all replicates. (B) In the set for BCAL0396 probe 9 was identified as an outlier. The heat shock and pre-heat shock signal intensities for this probe are lower than the majority, however the ratio is the same.

Figure 2 provides an example of the reproducibility of primary probes contained on the microarray. In figure 2a, all replicate probes of the gene BCAL0007 return similar signal intensity values. Moreover, the calculated Cy3/Cy5 ratios are nearly statistically identical with a coefficient of variation (CV) of 1.62%. Figure 2a is indicative of the majority of probe sets examined in this analysis (72 out of 117). Alternatively, we did identify a few cases of replicate probes that returned signal intensity values outlying those of their counterpart probes (figure 2b). In this probe set (BCAL0396), probe 9 is defined as an outlier. Notice, however that the Cy3/Cy5 ratios all lie within the same range (CV is 11.3%) and contribute the same up/down call for these probe replicates.

One advantage of using arrays amenable to dual-channel, competitive hybridization methods is the ability to minimize any differences in probe affinity. That is, since the observed signal intensity is a function of both probe affinity and target concentration, and since probe affinity is thought to be relatively constant over a range of target concentrations, then division of signal intensity values minimizes any affect of probe affinity on observed signal ratios. If the ratio of heat shock to pre-heat shock is greater than 1.5 the gene is considered to be up-regulated, if this ratio is below 0.7 the gene is down-regulated, and between these limits there is no significant change in gene expression. Of the primary probes, only 8 of 117 sets had one or more probes return ratios that provided different up/down calls than the majority. Moreover, only 2.4% of all primary probes had ratios that differed significantly from the rest of the subset. The majority of these discrepancies were probe replicates with ratios that were centered at the cut-off value (ratios ranging from 0.68–0.72). In fact, if the cut-off values are extended from 0.7 and 1.5 to 0.65 and 1.7 then only 2 probe sets of 117 had outlying probes.

Because each replicate contained the same sequence, the errors produced amongst primary probe sets were a result of experimental variations as well as spatial variations within each array. Given the excellent overall consistency of primary probes, less than 5% were identified as expression outliers or yielded different results when taking the ratio, it is clear that these experimental variations (i.e. hybridization, RNA preparation, etc.) and spot to spot variation are minimal for these arrays. In addition, outliers of up/down calls were only 2.4% suggesting that taking the ratio minimizes the effects of probe affinity. We next focused our efforts on examining variance associated with probes of alternative sequence directed against the same target gene.

### Alternative probe reproducibility

**Figure 3:**
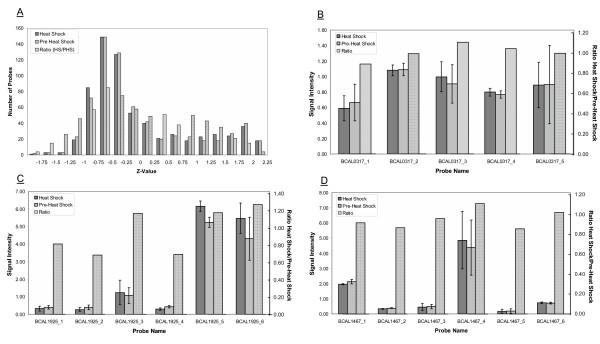
*Alternative Probes Sets*. (A) Z-values of heat shock, pre-heat shock and ratio probes. Most outliers were identified at the high end of each probe set. (B) Comparison of an alternative probe set (BCAL0317) with repeatable signal intensity and heat shock/pre-heat shock ratios. Ratio levels all return the same conclusion of no change in expression. (C) No probes were identified as signal intensity outliers in the set for BCAL1925, however both probes 2 and 4 contribute different probe conclusions (decrease in gene expression) than the majority of this set (no change in gene expression). (D) Conversely, in the set of alternative probes designed from BCAL1467 probe 4 is identified as an outlier, but error in signal intensity does not lead to incorrect up/down call.

We applied the same analysis described above for the primary probes to the entire probe set (primary and alternative) for a particular target. In this analysis primary probe replicates were represented by the mean of the whole set. Z-values were calculated for both heat shock and pre-heat shock gene expression (figure 3a) and Chauvenet's Criterion was applied to them. Based on this analysis, 67 of 117 probe sets produced a probe with outlier signals, 13 of which were primary probes. 9.5% of the total number of alternative probes returned signal values that were considered outliers. Recall that the primary probes had a 3.8% outlier rate due to natural variations (probe location, differences in binding, etc.). Under the assumption of additive error, at best only 6% error is due to differences in sequences. If this assumption cannot be made, however, the outlier rate is still less than 10%, which is sufficient for arrays (25% CV is considered good for spotted arrays, [8]). This surprisingly small number of outliers and can be linked to the high quality of Agilent arrays and the specificity of 60mer probes.

Figures 3b–3d provide examples of probe sets with reproducible values and sets that contained probes that returned outlier signal calls. The probe alternatives in figure 3b have very similar signal intensity and ratios that deliver the same conclusion of no change in gene expression. A probe set where no probes were deemed outliers (due to large overall standard deviation of the set) is shown in figure 3c, however probes BCAL1925_2 and BCAL1925_4 return different conclusions than the majority of probes in the set. Alternatively, in figure 3d probe BCAL1467_4 is identified as an outlier but the ratios of each probe concludes there is no change in expression. This is an example where outliers in magnitude of fluorescence are nullified by taking the ratio of the two dyes.

These results suggest that agreement of probe conclusion must also be considered as a measure of proper design (recall ratios greater than 1.5 are up-regulated genes, below 0.7 are down-regulated genes and ratios between 0.7 and 1.5 showed no change in expression). Of the total probe sets, 36 of 117 revealed at least one probe with a different conclusion than the rest. Of the 36 sets, 22 sets had only one differing probe, nine had two differing probes, and five were three-three splits. In addition, in 11 of these probe sets the primary probe was in the minority of gene expression conclusions (including the three-three splits). Including half the probes in a split set, about 7.8% of probes will produce a different gene expression finding than the majority of probes designed for each gene. Again, this is similar to the frequency of outliers and is a reasonable amount of error. However, as shown in figure 3, outliers do not necessarily produce faulty conclusions. In fact, only eight of the total 55 probes producing faulty conclusions were outliers. Therefore, being identified as an outlier in expression level does not necessarily contribute to a different conclusion. This further justifies the theory that taking the ratio of expression levels will minimize the effects of differing probe affinity. For gene expression analysis, errors in conclusions may be more important than actual signal intensity outliers. It appears that error is minimized if data analysis includes taking the ratio rather than by examining gene expression levels only.

### Prediction of outliers

The program used to design the probes includes subscores for four values: GC content, internal repeat, binding energy and self-annealment. The GC content subscore is strictly based on the percent of GC nucleotides in the oligo sequence, while the other subscores involve more complicated algorithms. Internal repeat compares the oligo sequence to its compressed version and the binding energy is of the oligo and its most homologous sequence. Finally, self-annealment predicts secondary structure due to self-annealment of the oligo [23].

**Figure 4:**
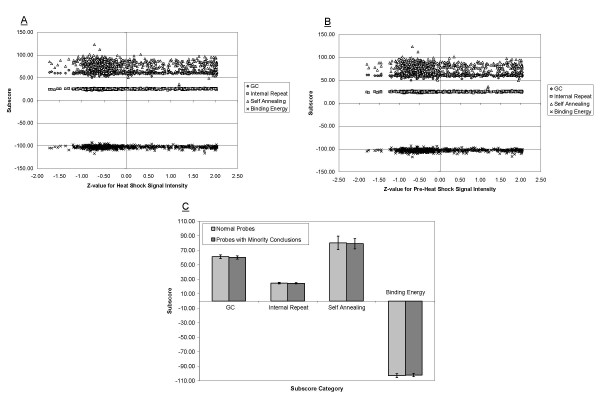
(A) Subscores (GC content, internal repeat, self-annealment and binding energy) are not predictive of outliers in heat shock signal intensity, (B) pre-heat shock signal intensity or (C) probes with minority conclusions.

Ideally, one could use these, or similar criteria, to predict probe performance without additional experimental data. However, our analyses indicate that there is no significant relationship between probe design subscore and z-value (probe performance) or subscore and expression conclusion for this array (figure 4). This result is not uncommon and is often noted as the inability to predict probe specificity without some preliminary experimental data [14].

## Conclusion

In this study, four major themes were examined: i) overall reproducibility of the arrays, ii) performance of primary probe replicates, iii) performance of alternative probes, and iv) predictive measures for bad probes. Overall the arrays appeared to be very reproducible; images of the arrays, including spot intensity and quality, were well replicated and probe performance between two replicates was the same. The high quality of the arrays endorses their application in bacterial studies. Primary probes also appeared to be reproducible within a single array with only about 3.5% identified as outliers. Alternative probes were slightly more problematic with closer to 10% outliers and 8% yielding different gene expression conclusions, yet these values are still within a tolerable amount of error for most microarray applications. Assuming independent probabilities of being identified as an outlier, including three probes against the same target would substantially reduce microarray error. It appears that analyzing the data using ratios of gene expression allows one to compensate for different probe affinities and non-specific binding. Since most two-color array studies compare an experimental condition in one dye channel to a control in the other, actual fluorescence/gene expression level is unimportant so long as the ratios of the two channels yields accurate and repeatable results. Since the percent of outliers identified while examining ratios is lower than the percent of outliers identified using signal intensity level, this appears to be a reasonable conclusion. Finally, factors used by this particular design program to select probes did not predict poorly performing probes.

## Methods

### Microarray probe and design

A summary of probe composition and purpose is included in table 1. We employed the ArrayOligoSel3.5 probe design program [23,24]. This program evaluates a number of different parameters, deemed important for designing effective oligonucleotide probes, and combines the results into a single score per probe. These parameters include i) uniqueness, ii) secondary structure, iii) complexity, iv) GC content, and v) distance from 3' end of ORF. The probes are then rank ordered by this score and used in the design of the array. The ArrayOligoSel3.5 program was run on the downloaded *B. cenocepacia *genome [25] with a total run time of approximately 10 hours (creating an output file of size 7.8 MB). 112 genes with duplicated sequences were excluded from the query. The scores and sequences for 10 candidate probes against the remaining 7113 genes were recorded. All probes were cross-checked against the human and mouse genome using the BLAST alignment tool. Only two probes displayed significant levels of identity. Probes from genomes other than *B. cenocepacia *were included to allow for cross-hybridization studies focusing on probe specificity and optimization of hybridization conditions. *Bacillus subtilis *control genes were selected to be identical to those contained on the Affymetrix *Pseudomonas aeruginosa *and *Escherichia coli *gene chips. The *P. aeruginosa *controls and *B. cenocepacia *control genes were selected based on their involvement in metabolism and known transcriptional regulation. The design of probes against *P. aeruginosa *and *B. subtilis *control genes were also performed as previously described.

### Strains and growth conditions

*B. cenocepacia *strain J2315 was used for all studies (provided by E. Mahenthiralingam). 10 ml of LB media was inoculated from bacterial freezer stock and incubated overnight at 37°C and 225 rpm. 50 ml of LB media was inoculated with 5 ml of the overnight culture and incubated. Bacterial growth was monitored until reaching a final OD_600_≈1.0. Samples were harvested by transferring 3 ml of culture to centrifuge tubes and aliquoted into heat shock and pre-heat shock samples. Pre-heat shock samples were briefly immersed (though not frozen) in liquid nitrogen to minimize RNA degradation. Samples were then centrifuged for 5 minutes at 5000 × g and 4°C. Following centrifugation, the supernatant was discarded and the bacterial pellets were immediately flash-frozen in liquid nitrogen and stored overnight at -80°C. Heat shock samples were placed in a 42°C water bath for 5 minutes. They were then incubated at 42°C and 225 rpm for 25 minutes before cells were flash frozen and harvested as previously described above.

### RNA preparation and cDNA synthesis

Total RNA was extracted from all bacterial pellets using the Rneasy Mini Kit and the QiaShredder digestion column (Qiagen, Valencia CA). On-column digestion with DNAseI was employed to minimize genomic DNA contamination. Following treatment on the Rneasy Mini Column, RNA was further purified by ethanol precipitation, which was found to improve cDNA yield. To quantify the amount of RNA present, OD_260 _and OD_280 _readings were taken of each solution using a UV-Vis spectrophotometer (UV Mini 1240, Shimadzu). All RNA samples displayed A_260/280 _ratios greater than 1.9 and clear rRNA bands were observed following agarose gel electrophoresis.

Both cDNA synthesis and labeling were performed using CyScribe Post-Labeling Kit according to manufacturer's instructions (Amersham Biosciences, Piscataway, NJ). Anchored oligo(dT) was not added to the primer annealing in order to retain bacterial RNA for further analysis. Approximately 10 μg RNA was labeled by reverse transcription with either Cy3-dCTP or Cy5-dCTP. Following the degradation of mRNA, amino allyl modified cDNA was purified with an ethanol precipitation, as outlined in the Amersham protocol. AutoSeq G-50 Spin columns (Amersham Biosciences) were used to purify the fluorescently labeled cDNA. In order to calculate the quantity of cDNA produced and dye incorporation, the spectrophotometer was used as according to the manufacturer's protocol.

### Microarray hybridization, washing, and scanning

For all arrays, the In Situ Hybridization Kit Plus and associated protocols were used (Agilent Technologies, G2530-40001). Microarrays were prepared in Agilent Technology's Hybridization Chamber according to manufacturers instructions (G2530-60001). For improved hybridization it was necessary to add an additional 15 μL of 2 × hybridization buffer (included in the In Situ Hybridization Kit Plus) to the hybridization mix. Once loaded into the hybridization chamber, samples were placed in the hybridization oven (Agilent Technologies, G2505-80085) and incubated for 17 hours at 65°C while rotating at setting 5. Following hybridization, samples were washed according to Agilent's wash procedure. Microarrays were scanned using Agilent Array scanner (G2565AA). Following scanning, array images were analyzed with Image Analysis ver. A.6.1.1. Hybridizations were performed in triplicate.

### Data analysis

Data was normalized by dividing the difference between signal and background signal by the average signal for the respective fluorophore. A probe set was defined as all probes designed for a single gene including a primary probe and 4–5 alternatives with differing sequences. Primary probes included 9–15 replicates of the same probe sequence. All probes were compared to the average and standard deviation of their probe set.

Z-values for primary probes were calculated using the following equation:

z = (signal_probe _- average signal_replicates_)/σ_replicates_

Z-values for alternative probes were calculated using the same formula and substituting the average and standard deviation of the whole set (for each set, the average of the primary probe replicates was used for the primary probe value). This analysis was carried out for pre-heat shock and heat shock expression levels independently.

A semi-quantitative measure was also used to examine consistency of probe conclusion (of interest in gene expression analysis). Ratios of heat shock to pre-heat shock signal intensity greater than 1.5 were classified as up-regulated, lower than 0.7 were labeled down-regulated, and no change in gene expression was identified between 0.7–1.5.

## Authors' contributions

Danielle Leiske: Responsible for the majority of data analysis and writing of the article.

Anis Karimpour-Fard: Assistance with data analysis and article editing.

Patrick Hume: Performed experiments on which data analysis was based and wrote the majority of the methods section.

Ben Fairbanks: Performed preliminary experiments optimizing hybridization conditions for this particular array.

Ryan T. Gill: PI for this project- responsible for all aspects of the research.

## Figures and Tables

**Table 1:** Probe distribution and purpose for *B. cepacia *8,400 probe array design.

**Probe Identity**	**Probes**	**Purpose**
*B. cenocepacia *genes	6786 (5101 genes)	Transcriptional profiling
Replicate probes	1178	Determine inter-probe variance
Alternative probes	558	Determine intra-probe variance
*P. aeruginosa *PAO1 genes	986 (50 genes)	Assess probe specificity and optimize hybridization conditions
Positive controls	246	Troubleshooting and normalization/quantification
Agilent controls	386	Manufacturers requirement

Totals	8455 (5151 genes)	
